# Whole Genome and Global Gene Expression Analyses of the Model Mushroom *Flammulina velutipes* Reveal a High Capacity for Lignocellulose Degradation

**DOI:** 10.1371/journal.pone.0093560

**Published:** 2014-04-08

**Authors:** Young-Jin Park, Jeong Hun Baek, Seonwook Lee, Changhoon Kim, Hwanseok Rhee, Hyungtae Kim, Jeong-Sun Seo, Hae-Ran Park, Dae-Eun Yoon, Jae-Young Nam, Hong-Il Kim, Jong-Guk Kim, Hyeokjun Yoon, Hee-Wan Kang, Jae-Yong Cho, Eun-Sung Song, Gi-Ho Sung, Young-Bok Yoo, Chang-Soo Lee, Byoung-Moo Lee, Won-Sik Kong

**Affiliations:** 1 Department of Biomedical Chemistry, Konkuk University, Chung-Ju, Republic of Korea; 2 Macrogen Inc., Seoul, Republic of Korea; 3 Department of Life Sciences and Biotechnology, Kyungpook National University, Daegu, Republic of Korea; 4 Graduate School of Biotechnology and Information Technology, Hankyong National University, Ansung, Republic of Korea; 5 Department of Pharmaceutical Engineering, Sangji University, Wonju, Republic of Korea; 6 National Academy of Agricultural Science, Rural Development Administration, Suwon, Republic of Korea; 7 Mushroom Research Division, National Institute of Horticultural and Herbal Science, Rural Development Administration, Eumsung, Republic of Korea; 8 Genomic Medicine Institute (GMI), Medical Research Center, Seoul National University, Seoul, Republic of Korea; AIT Austrian Institute of Technology GmbH, Austria

## Abstract

*Flammulina velutipes* is a fungus with health and medicinal benefits that has been used for consumption and cultivation in East Asia. *F. velutipes* is also known to degrade lignocellulose and produce ethanol. The overlapping interests of mushroom production and wood bioconversion make *F. velutipes* an attractive new model for fungal wood related studies. Here, we present the complete sequence of the *F. velutipes* genome. This is the first sequenced genome for a commercially produced edible mushroom that also degrades wood. The 35.6-Mb genome contained 12,218 predicted protein-encoding genes and 287 tRNA genes assembled into 11 scaffolds corresponding with the 11 chromosomes of strain KACC42780. The 88.4-kb mitochondrial genome contained 35 genes. Well-developed wood degrading machinery with strong potential for lignin degradation (69 auxiliary activities, formerly FOLymes) and carbohydrate degradation (392 CAZymes), along with 58 alcohol dehydrogenase genes were highly expressed in the mycelium, demonstrating the potential application of this organism to bioethanol production. Thus, the newly uncovered wood degrading capacity and sequential nature of this process in *F. velutipes*, offer interesting possibilities for more detailed studies on either lignin or (hemi-) cellulose degradation in complex wood substrates. The mutual interest in wood degradation by the mushroom industry and (ligno-)cellulose biomass related industries further increase the significance of *F. velutipes* as a new model.

## Introduction

*Flammulina velutipes*, also known as winter mushroom or Enokitake, belongs to the Marasmioid clade [1] (part of the order Agaricales), which includes the recently sequenced model mushroom *Schizophyllum commune* [2] (split gill fungus) and the edible mushroom *Lentinula edodes* (Shiitake). *F. velutipes* is further classified in the family Physalacriaceae, together with *Armillaria* species (honey fungus), which encompass edible fungi and plant pathogens. As such, the genome of *F. velutipes* KACC42780 is significant within *Flammulina* species and in studies of other edible and environmentally important mushrooms.

*F. velutipes* is one of the 6 most actively cultivated mushroom species in the world; over 300,000 tons of this mushroom are produced per year [3]. As a typical white-rot fungus, *F. velutipes* can be found on dead wood. Its distribution is limited to the temperate zones of the world because a cold period is required for fruiting [4]. This behavior is reflected in commercial cultivation, which uses media mimicking wood (sawdust-based or corncob) and cold treatment. Reduction of the crop cycles (45–55 days), cold treatment, and the amount of waste material (1 kg substrate/300 g mushrooms) are important topics for modern cultivation.

Bioethanol production has been attracting worldwide interest due to global warming and the need for global energy security. Lignocellulose from plant cell walls, the most abundant biomass source in nature, is expected to have applications in future bioethanol production. Moreover, the edible mushroom *F. velutipes* has been shown to be an efficient ethanol producer, and its properties of ethanol fermentation from various sugars and whole crop sorghums have been characterized in several studies [5], [6]. However, the production of ethanol from lignocellulosic biomass is expensive; thus, many researchers are focusing on low-cost consolidated bioprocessing (CBP) for bioethanol production. Bioethanol could be produced in one reactor by CBP, which includes the following 3 biological events: production of lignocellulose-degrading enzymes, hydrolysis of polysaccharides present in pretreated biomass, and fermentation of hexose and pentose sugars [7]. Basidiomycetes can degrade lignin [8], and some basidiomycetes produce alcohol dehydrogenase, allowing the production of ethanol using mushrooms instead of *S. cerevisiae* [9], [10]. These properties of basidiomycetes appear suitable for use in new strategies of bioethanol production. However, the use of basidiomycetes in bioethanol production is not common, and the ethanol fermentation abilities of basidiomycetes are not well characterized. Therefore, better insight into the substrate degrading machinery of *F. velutipes* will be helpful.

Although *F. velutipes* can be cultured on defined media and can be genetically modified by transformation methods, such as Agrobacterium-mediated transformation (AMT), the calcium-PEG method [5], research identifying the genetic sources involved in these various functions is restricted because only few genes are known. In order to successfully apply multifaceted approaches (combining mycological, biochemical, pharmacological, and metabolic technique studies), identification of the whole genome sequence of *F. velutipes*, in addition to subsequent gene expression profiling, will be necessary to understand the genetic basis of mushroom formation and regulation of developmental stages and tissue growth.

Here, we report the genome sequence of *F. velutipes*. This information, along with predicted genes of *F. velutipes*, will facilitate our understanding of the fundamental and specific cellular processes of mushroom-forming basidiomycete fungi for commercial production by molecular breeding and industrial use, such as bioethanol production from biomass.

## Material and Methods

### Strains and Culture Conditions

*Flammulina velutipes* was obtained from the Korean Agricultural Culture Collection (KACC; Rural Development Administration, Korea; http://www.genebank.go.kr/) and was grown at 26°C on MCM agar (0.2% peptone, 2% glucose, 0.2% yeast extract, 0.05% MgSO_4_, 0.046% KH_2_PO_4_, 0.1% K_2_HPO_4_, and 1.5% agar) for two weeks. *F. velutipes* KACC42780 monokaryotic strain and *F. velutipes* KACC43778 dikaryotic strain were used for genome sequencing and transcriptome analysis, respectively. The *F*. *velutipes* dikaryotic strain KACC43778 resulted from a cross between strains KACC42780 and KACC43777. For genomic DNA and total RNA isolation from mycelia, a 300-mL Erlenmeyer flask containing 50 mL MCM medium was inoculated with fresh plugs from the plate (five mycelial plugs/flask) and incubated at 26°C for two weeks without agitation. To obtain primordia and fruiting bodies, mycelial plugs were inoculated on a growth medium consisting of 80% poplar sawdust, 20% rice bran, and 65% water in a 1,000 cm^3^ disposable bottle after sterilization [11]. Inoculated bottles were incubated at 20°C under dark conditions for 25 days, and then transferred to fruiting conditions, maintained at 15°C, at 90% humidity, and with continuous light. Primordia, which measured 2–3 mm in diameter, budded at 10 days after physical stimulation and were collected. After budding of primordia, fruiting was stimulated by cold shock at 4°C for 3 days. At 7 days after cold stimulation, fruiting bodies were matured in conditions of 9°C and 75% humidity and collected.

### Genomic DNA Preparation

For isolation of genomic DNA, 400 μL of Soil DNA Extract buffer (100 mM NaCl, 50 mM EDTA, 0.25 M Tris-HCl, 5% SDS), 400 μL of 2×CTAB buffer (2% CTAB, 100 mM Tris-HCl pH 8.0, 20 mM EDTA pH 8.0, 1.4 M NaCl, 1% polyvinyl pyrrolidone), and 500 μL phenol-chloroform-isoamylalcohol (25∶24∶1, Bioneer, Korea) were added to 0.1–0.5 g lyophilized or fresh mycelium and briefly vortexed. After 5 min of incubation at room temperature, samples were centrifuged at 13,000 rpm at 4°C, for 5 min. Supernatants were mixed with 0.7 volumes isopropanol and centrifuged for 10 min at 4°C. After washing with 70% ethanol, air dried samples were eluted in 50–100 μL TE and treated with RNase A (Bio Basic Inc, Canada) for 30 min at 60°C.

### Genome Sequencing and Assembly

Genome Sequencer FLX system (GS-FLX Titanium, Life Sciences) libraries were generated using genomic DNA from *F. velutipes* strain KACC42780 in accordance with the manufacturer’s instructions. For GS FLX Titanium paired-end sequencing, 15 ug of genomic DNA was sheared into DNA fragments ranging from 400 to 800 bp by nebulization. After both ends of the DNA fragments were repaired and phosphorylated, 2 types of adaptors (A and B) were ligated to the DNA fragments. DNA fragments carrying the 5′-biotin of adaptor B from the ligation mixture were immobilized onto magnetic streptavidin-coated beads. Single-stranded template DNA (ssDNA) molecules carrying adaptor A at the 5′-end and adaptor B at the 3′-end were isolated by alkaline denaturation. These purified ssDNAs were then hybridized to DNA capture beads and clonally amplified by an emulsion polymerase chain reaction (PCR) method. After denaturation of the amplified double-stranded DNAs on the capture beads, beads with single-stranded molecules were spread onto each well of a pico titer plate. For GS FLX Titanium mate-paired library sequencing, 15 ug of genomic DNA was sheared into DNA fragments (3 and 5 kb). After the ends of the DNA fragments were repaired and internal adaptors were ligated to the DNA fragments, each DNA fragment was circularized and self-ligated. The circular DNA was sheared into DNA fragments ranging from 400 to 800 bp by nebulization. DNA fragments carrying the 5′-biotin of the internal adaptor from the ligation mixture were immobilized onto magnetic streptavidin-coated beads. Mate-paired sequencing was carried out in a method similar to that described above. Approximately 3,204,100 (37.2×), 1,392,885 (15.6×), and 1,294,191 (10.9×) reads from the GS FLX single end, 3 and 5 kb mate libraries, respectively, were assembled using Newbler Assembler (v 2.5.3) into 3,581 contigs (≥500 bp) of 35.34 Mb with N50 and average contig lengths of 147,615 and 9,867 bp, respectively. Finally, 271 scaffolds were formed with N50 of 436,467 bp (average of 126,275 bp).

### BAC Library Construction and Physical Mapping

To generate a physical map, the BAC library was prepared from a partial *Hind*III and *EcoR*I digest of high-molecular-weight genomic DNA from *F*. *velutipes* in the vector pBeloBAC11 using standard methods [12]. BAC clone DNA was extracted from a single colony by a standard alkaline lysis extraction method. Inserts of 7,680 BAC clones were sequenced from both ends using universal primers, an ABI 3730×l DNA analyzer, and an ABI BigDye Terminator Cycle Sequencing Kit (Applied Biosystems, Foster City, CA, USA). A physical map of *F*. *velutipes*, based on BAC clones, was constructed by high information content fingerprinting (HICF) analysis according to the manufacturer’s standard protocol (ABI SNaPShot Multiplex System). BAC clones were analyzed in terms of band sizes following restriction enzyme digestion by GeneMapper and Genoprofiler, which were then assembled into an FPC map by FPC V8. The summed length of 13 FPC contigs was 39,612 consensus bands (CB), very close to the sum of contig sequence lengths [13].

### Gap Filling and Scaffolding

The 272 BAC clones, spanning the minimal tiling path in the FPC map but not covered by contig sequences, were selected and fully sequenced. The 25×∼35× GS-FLX shotgun reads were assembled by Newbler Assembler (v 2.5.3) software into corresponding draft BAC sequences for 161 BAC clones, while 8×∼12× Sanger shotgun reads were assembled by Phrap software for 111 BAC clones. The sequencing gaps in the draft assemblies of each BAC clone were filled by primer walking. The resulting 272 finished BAC clone sequences were assembled by Phrap together with the contigs from the 271 initial scaffolds, producing 11 final scaffolds, with N50 being 3.9 Mb (500 scaffold contigs spanning a total of 35.3 Mb). Each of the 11 scaffolds was confirmed to be the corresponding chromosome.

### Scaffold Assignment

We previously reported the electrophoretic karyotype of the *F*. *velutipes* KACC42780 monokaryotic strain using pulse field gel electrophoresis (PFGE) [14]. Therefore, PFGE and Southern hybridization were used to assign the 11 scaffolds to chromosomal bands. The scaffold primers used to make DNA probes were designed to generate unique DNA fragments of 300 bp by PCR amplification. A DNA probe was generated for each of the 11 scaffolds representing chromosome pieces. PFGE and Southern hybridization analysis for assignment of scaffolds to chromosomes were carried out according to methods described by Park et al. [14].

### Gene Modeling and Annotation

Gene identification was carried out using several methods, including *ab initio* gene structure prediction (Fgenesh; http://www.softberry.com), a homology-based approach (Fgenesh+; http://www.softberry.com), and transcriptome-based gene identification (Cufflinks; http://cufflinks.cbcb.umd.edu/manual.html). Fgenesh trained with *Coprinopsis cinerea* produced 10,105 gene models. Fgenesh+ trained for *C*. *cinerea* and *Laccaria bicolor* produced 13,544 and 20,614 gene models, respectively. Cufflinks was used to assemble Illumina transcriptome reads with the parameter set at “–max-intron-length 5,000”; 11,324,134 paired-end reads produced 10,528 gene models.

In order to remove redundancy of the genes, the following rules were used. Firstly, Fgenesh+ models with a score of 100 or greater were collected, and the ones from *C*. *cinerea* were kept when there were any overlaps between *C*. *cinerea*- and *L*. *bicolor*-based models (gene set 1, containing 6,283 genes). The gene models from Cufflinks were then selected and included in gene set 1, forming gene set 2 (8,082 genes) when they did not overlap with the models in gene set 1 and their protein coding region covered at least 50% of their transcripts. The reason for this complicated rule was that Cufflinks produced many transcripts with frameshift mutations, resulting in truncated protein products. Finally, the additional 3,204 gene models from the Fgenesh prediction were included when they did not overlap with the models in gene set 2, resulting in the final gene set 3 (12,218 genes). The overlaps between gene models were checked by bedtools [15]. For functional annotation of the predicted genes, the genes were compared using BLAST (version 2.2.17) software with a series of protein and nucleotide databases, including the NCBI nucleotide (Nt; http://blast.ncbi.nlm.nih.gov/Blast.cgi), nonredundant set (Nr; http://blast.ncbi.nlm.nih.gov/Blast.cgi), UniProt/Swissprot (http://www.ebi.ac.uk/swissprot/sptr_stats/index.html and http://www.expasy.org/sprot/relnotes/relstat.html), Gene Ontology (GO) [16], eukaryotic orthologous groups (KOGs) [17], CDD, and Kyoto Encyclopedia of Genes and Genomes (KEGG; http://www.genome.jp/kegg/) protein databases. The E-value cutoff for BLAST comparison was 0.001. The genome contained 287 tRNAs, as predicted by tRNAscan-SE (version 1.23) [18] and 10 pseudogenes.

### Orthologs of *F*. *Velutipes* Proteins

The predicted genes (proteins) were clustered into orthologous groups using OrthoMCL version 2.0 software with default parameters [19]. Orthologs from *Aspergillus nidulans* [20], *C*. *cinerea* [21], *Cryptococcus neoformans* [22], *L*. *bicolor* [23], *Neurospora crassa* [24], *Phanerochaete chrysosporium* [25], *Postia placenta* [26], *Saccharomyces cerevisiae*, [27], *S*. *commune* [2], and *Ustilago maydis* [28] were analyzed. All-versus-all protein comparison was accomplished using the BLASTP module of NCBI BLAST software (version 2.2.20) with E-value cutoff at 0.001. Based on the OrthoMCL analysis, 13,429 orthlog groups were identified with 323 members in the largest group.

### Cazyme Annotation

The search and functional annotations for carbohydrate-active modules and ligninolytic enzymes, including glycoside hydrolases (GHs), glycosyltransferases (GTs), polysaccharide lyases (PLs), carbohydrate esterases (CEs), and auxiliary activities (AAs; formerly FOLymes), were performed using the dbCAN CAZyme annotation program (http://csbl.bmb.uga.edu/dbCAN/) [29] with default parameters and the Carbohydrate Active Enzymes (CAZy) database (http://www.cazy.org).

### Repeat Content Identification

RepeatMasker (A.F.A. Smit, R. Hubley, & P. Green, unpublished data; current version: open-3.3.0 at http://repeatmasker.org) was used to generate *de novo* repeat sequence predictions for *F. velutipes* with default parameters. Transposable elements (TEs) and repetitive elements were further identified by comparison to known repeats with Censor (http://www.girinst.org/censor/index.php) with default parameters.

### Total RNA Preparation

Samples from three biological replicates of each developmental stage and tissue were ground to a fine powder under liquid nitrogen using a mortar and pestle and stored at −80°C. Total RNA was prepared from tissue samples (100 mg) using TRIzol reagent in accordance with the manufacturer’s instructions (Invitrogen Life Technologies, USA). Total RNA (10 μg) was treated for 30 min at 37°C with 1 U of RQ1 RNase-free DNase (Promega, USA).

### Transcriptome Analysis

For comparative transcriptome analysis, total RNA was isolated from 3 different developmental stages of the *F. velutipes* KACC43778 dikaryotic strain as described [30]. Total RNA was further processed using an Agilent Technologies 2100 Bioanalyzer for RNA integrity and quality (value greater than or equal to 8). Library preparation and sequencing were performed with 1 μg of each total RNA using a HiSeq2000 sequencing system under the manufacturer’s standard protocol. Short reads of the *F. velutipes* KACC43778 dikaryotic strain (a total of 11,324,134 reads) were assembled using Cufflinks (http://cufflinks.cbcb.umd.edu/manual.html). The 10,528 transcripts were used in the following steps. Short-reads (a total of 11,324,134 reads) were directly mapped to the 12,218 predicted genes of the *F*. *velutipes* KACC42780 genome using BWA (Burrows-Wheeler Aligner) [31] with the parameter set at -q 20 (-q: quality threshold for read trimming down to 35 bp), and the Genome Analysis Toolkit (GATK) [32] was applied based on quality score recalibration using standard hard filtering parameters. Mapping results were manipulated alignments and counting reads of mapping by samtools [33] (http://samtools.sourceforge.net/), and the number of aligned reads was interpreted as the expression level (reads per kilobase of exon model per million mapped reads [RPKM]). In addition, we performed quantile normalization using the R package ‘preprocesscore’ [34] within each sample.

### RT-PCR Analysis

For RT-PCR analysis, the reverse transcription of RNA (1 μg) in a 20-μL reaction volume was performed using oligo-dT18 and ImProm-II reverse transcriptase (Promega, USA). Reactions were incubated at 25°C for 5 min, at 42°C for 60 min, and then at 70°C for 10 min to inactivate the reverse transcriptase. The PCR reaction was conducted in a 50-μL reaction mixture containing 10 mM dNTP mixture, 10 pmol of each specific primer, 1 unit Taq-polymerase (TaKaRa Korea Biomedical Inc., Seoul, Korea), 10×PCR buffer (100 mM Tris-Cl, pH 8.3, 500 mM KCl, and 25 mM MgCl_2_), and 1 μL cDNA product. PCR was performed with the primers ctg01_AA_00276 (5′-CTGCAACAGCACCCAATCTA-3′ and 5′-CTAGAGGGCGACGTTGAGAG-3′), ctg26_AA_00146 (5′-CTGCTACCATCCTCGCTTTC-3′ and 5′-ATGCGTCACAGTTGCTGTTC-3′, ctg26_AA_00173 (5′-CAGGTCAAGTTCTCGGCTTC-3′ and 5′-GCCAAAGAGCGTAGTCGTTC-3′), and ctg03_AA_00481 (5′-CCACCAAGAACGGACAAGTT-3′ and 5′-TGGCTTGTAATGTGCCAGAG-3′).

### Data Access

This whole-genome sequencing project has been deposited at DDBJ/EMBL/GenBank (http://www.ncbi.nlm.nih.gov/) under the accession number AQHU00000000 (the project accession number PRJNA191921). The version described in this paper is the first version. Sequence reads have been deposited in the short-read archive at GenBank under the following accession numbers: SRA068777 contains genomic reads by the Genome Sequencer FLX system (GS-FLX Titanium) and SRA068291 contains RNA-Seq reads by the Illumina HiSeq2000. All of the data generated in this project, including those related to gene prediction, gene functional annotations, and transcriptomic data, are available on our interactive web DB (http://112.220.192.2/fve/).

## Results

### General Features of the Genome

**Figure 1:**
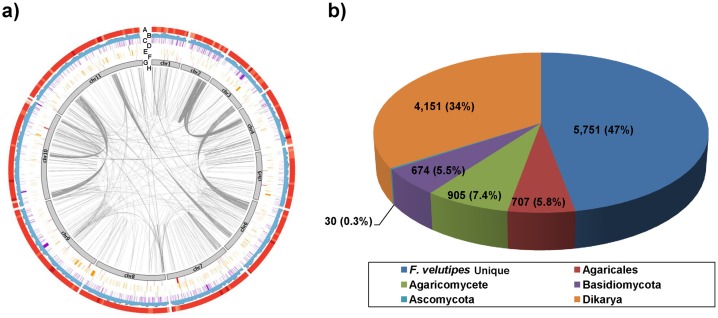
The *F. velutipes* genome map (a). A, GC content was calculated as the percentage of G+C in 100-kb non-overlapping windows. B, Gene density is represented as the number of genes in 100-kb non-overlapping windows. C, Transcription factors. D, Distribution of alcohol dehydrogenase genes. E, Distribution of CAZyme genes. F, Distribution of AA genes. G, Pseudochromosomes, represented clockwise starting from center above. Blocks represent 11 *F. velutipes* pseudochromosomes. Numbers indicate the chromosome number. H, Genome duplication: regions sharing more than 90% sequence similarity (e-value ≤1e-100) and more than 80% query coverage are connected by grey lines. (b) Presence of orthologs of the predicted *F. velutipes* genes in fungal taxons (right). Detailed information is given in Tables S1 and S6.

**Figure 2:**
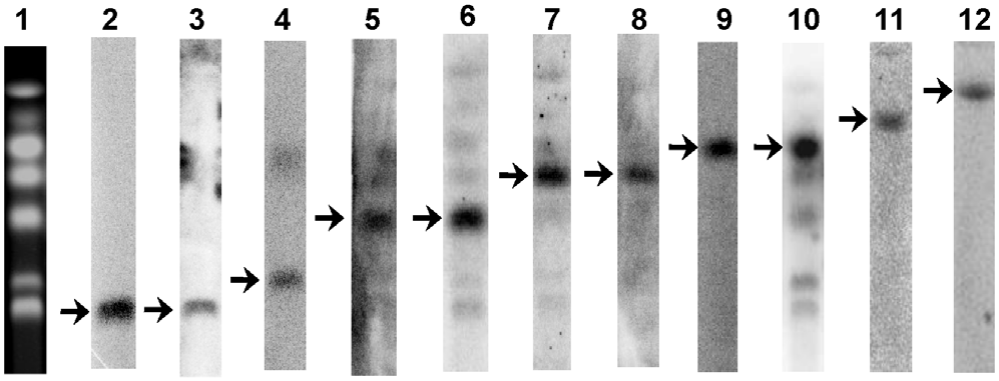
Scaffold assignments by Southern hybridization analysis with CHEF-separated chromosomes. A DNA probe was generated for each of the 11 scaffolds representing chromosome regions. Arrows indicate the positive signals. 1, *F. velutipes* KACC42780 electrophoretic karyotype; 2, ctg26 probe (chromosome 1); 3, ctg02 probe (chromosome 2); 4, ctg01 probe (chromosome 3); 5, ctg06 probe (chromosome 4); 6, ctg29 probe (chromosome 5); 7, ctg13 probe (chromosome 6); 8, ctg33 probe (chromosome 7); 9, ctg03 probe (chromosome 8); 10, ctg11_1 probe (chromosome 9); 11, ctg11_2 probe (chromosome 10); 12, ctg05 probe (chromosome 11).

The genome sequence of the *F. velutipes* monokaryotic strain KACC42780 was analyzed by the Genome Sequencer FLX system (GS-FLX Titanium) with 37.7× (paired-end) and 60× (mate-paired; 3 and 5 kb) sequence coverage (**Figure S1**). The Newbler Assembler (v 2.5.3) program was used to assemble the draft genome sequence. The resulting assembly consisted of 500 sequence contigs with a total length of 35.3 Mb and an N50 length of 252.7 kb (that is, 50% of all bases were contained in contigs of at least 252.7 kb). To cover the gaps in the draft genome assemblies resulting from the Newbler Assembler, 272 BAC clones (minimal tiling paths) were selected and mapped on the draft genome assemblies. After anchoring the BACs, contigs were assembled into 11 largest scaffolds, representing the 11 chromosomes [14], with a total length of 35.6 Mb (including gaps between contigs) and an N50 length of 3.9 Mb (**Figure 1** **)**. PFGE and Southern hybridization analysis allowed 11 scaffolds to be assigned to each corresponding chromosome of *F. velutipes* (**Figure 2**). The general features of the *F*. *velutipes* genome, including assembly and gene model statistics, are presented in **Table 1** and **Table S1**. The genome contained 12,218 predicted protein-encoding genes, of which 86% were supported by expressed sequenced tags (ESTs) and 91.5% by RNA-seq (**Tables S2** and **S3**). However, 1,451 out of the 12,218 models were shorter than 500 nt in size, and therefore, most of these regions were unlikely to encode proteins. The total number of genes in *F*. *velutipes* was comparable to that of its nearest sequenced species, *S*. *commune* [2], as well as to other basidiomycetes of similar genome size (**Table 2**). In addition, 287 tRNA genes in the *F*. *velutipes* genome were identified by tRNAscan-SE [17] (**Table S4**). The average exon and intron lengths were 245.7 and 180.4 nucleotides, respectively. The average gene density, similar among the larger scaffolds, was 1 gene per 2.9 kb (**Table S1**). Of the 12,218 predicted genes, 74% (9,051) had significant sequence similarity to documented proteins in BLAST-NR (**Table S5**). BLASTP searches against the NCBI-NR fungal protein database revealed that 7,169 (58.6%) of the predicted proteins shared sequence similarity (BLAST-NR) to documented fungal sequences (**data not shown)**. Cluster analysis with other sequenced fungi identified 5,748 groups containing at least 1 *F*. *velutipes* protein (**Table S6**). Analysis of these clusters suggested that 34% of *F*. *velutipes* proteins had orthologs amongst the Dikarya and were thus conserved in Basidiomycota and Ascomycota (**Figure 1**). No less than 47% (5,751) of genes were unique to *F*. *velutipes*, according to OrthoMCL analysis, of which 46.7% (2,688) of genes had inparalog(s) in *F*. *velutipes*. Of the 12,218 predicted genes, 50.8% (6,214) and 69.9% (8,543) matched with unknown and known functions (cut-off *e*-value≤0.001) and could be annotated with a gene ontology (GO) [16] term and functional catalog (FunCat), respectively (**data not shown**). In addition, 9,589 (78.48%) and 9,049 (74%) proteins showed sequence similarity to sequenced organisms in the KOGs [16] (eukaryotic orthologous groups) and KEGG (Kyoto Encyclopedia of Genes and Genomes, http://www.genome.jp/kegg/) databases, respectively (**data not shown**). In addition, the *F*. *velutipes* genome contained genes encoding 681 putative transcription factors (**Table S7**).

**Table 1:** General features of the *F*. *velutipes* genome.

	Scaffold Total	11.0
	Scaffold Sequence Total	35.6 Mb
	Scaffold N50	3.9 Mb
	Scaffold L50	3.9 Mb
Sequence and assembly	Contig Total	500.0
statistics	Contig Sequence Total	35.3 Mb
	Contig N50	252.7 kb
	Contig L50	252.7 kb
	Estimated Depth	60×
	ESTs mapped to scaffolds	86.0%
	GC content	48.99%
	Protein coding genes	12,218
	Gene Length (bp)	2,294
	Transcript Length (bp)	1,425
	Protein Length (aa)	453
Gene model statistics	Exons per Gene	5.8
	average exon Length (bp)	245.7
	average intron Length (bp)	180.4
	Genes per Mb Scaffold	342.8
	tRNAs	297

**Table 2:** Comparison of genome characteristics between *F*. *velutipes* and other basidiomycetes.

Genome characteristics	*F*. *velutipes*	*S*. *commune*	*L*. *bicolor*	*C*. *cinerea*	*P*. *chrysosporium*	*U*. *maydis*
Strain	KACC42780	H4–8	S238N–H82	Okayama7#130	RP78	521
Sequencing institution	RDA, Macrogen Co.	JGI	JGI	Broad	JGI	Broad
Genome assembly (Mb)	35.6	38.5	64.9	37.5	35.1	19.7
Number of protein-coding genes	12,218	13,181	20,614	13,544	10,048	6,522
GC contents (%)	48.99	56.6	46.6	51.6	53.2	54.0
Average gene length (bp)	2,294	1,794.9	1,533.0	1,679.0	1,667.0	1,935.0
Average coding-sequence length (bp)	1,425	1,430.3	1,134.0	1,352.0	1,366.0	1,840.0
Average exon length (nt)	245.7	249.3	210.1	251.0	232.0	1,051.0
Average intron length (nt)	180.4	79.0	92.7	75.0	117.0	127.0

### Global Gene Expression

**Figure 3:**
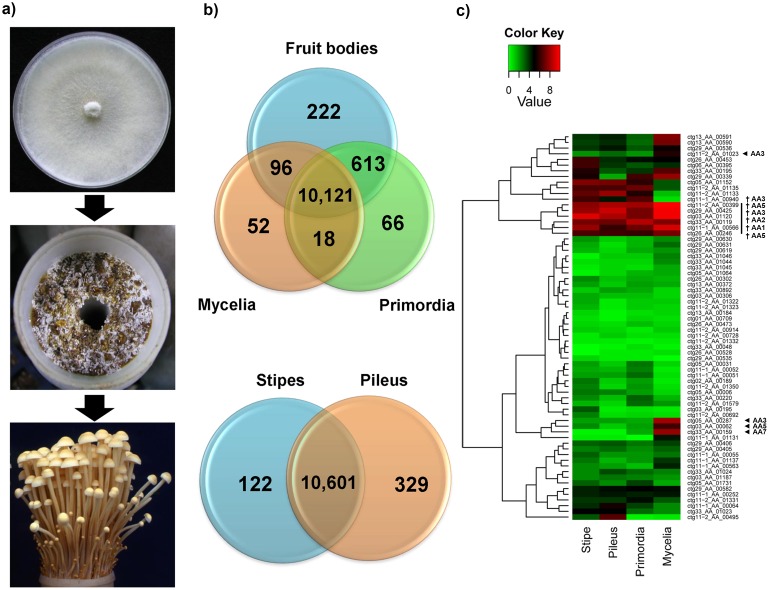
Comparative analysis of gene expression at 3 different developmental stages and in different tissues of *F. velutipes*. (a) The 3 developmental stages of *F. velutipes*. (b) The Venn diagram shows unique and common genes expressed across the different developmental stages and the different tissues. (c) Heat map of expression of AA genes at 3 developmental stages and in different tissues of *F. velutipes*. The bar at the top of the panel represents the expression values (log2 based normalization value). AA1, multicopper oxidase (laccases, ferroxidase, laccase-like multicopper oxidase); AA2, classII peroxidase (manganase peroxidase, lignin peroxidase, versatile peroxidase); AA3, GMC oxidoreductase (cellobiose dehydrogenase, aryl-alcohol oxidase/glucose oxidase, alcohol oxidase, pyranose oxidase); AA5, radical-copper oxidase (glyoxal oxidase, galatose oxidase); AA7, glucooligosaccharide oxidase. ◂ indicates a 2-fold increase in gene compared to the next highest expression level for a particular stage or tissue and exhibiting peaked expression (≥5.5 log2 values) during the mycelial stage. † indicates that the AA gene showed a higher expression level than the overall average expression level of all other AA genes during all stages and in all tissues. Detailed information is given in Table S10.

NGS-based RNA-Seq was carried out to assess gene expression in 3 key developmental stages in *F. velutipes*: mycelium, primordium, and mature fruiting bodies, the latter which was subdivided into the 2 major tissues that make up mature fruiting bodies, i.e., stipe and pileus (**Figure 3** **and Table S3**). This revealed high numbers of expressed genes in all stages of *F. velutipes*. A total of 11,188 genes were expressed at least once, with 10,121 genes expressed at all stages (**Figure 3b**). Only 1,030 genes (8.4%) were never expressed under the examined conditions. Our data further showed the first global expression analysis linked to the complete genome of *F. velutipes*. These data revealed that fruiting bodies of *F. velutipes* were highly specialized, with 2% of the genes being stage specific, compared to 0.6% and 0.5% for primordia and mycelia. Primordia and complete fruiting bodies (stipe and pileus) also showed more specific genes in common (613) than fruiting bodies and mycelia (96 genes) or primordia with mycelia (18 genes), indicating more overlap in developmental processes taking place in primordia and mature fruiting bodies. Within fruiting bodies, a high level of differentiation between the distinct tissues also became apparent. The differences between the stipe and pileus (122 and 329 tissue-specific genes) implied a higher level of specification in pilei, but demonstrated that stipes, though superficially simple, are subjected to complex regulation as well. This is illustrated by the activity of mitochondria, which was reduced in stipe tissue (**Table S8**), but not in pilei. Two hundred four of the 222 fruiting body-specific genes were unique to *F*. *velutipes*. Eighteen showed orthology with Agaricales or more distant fungi, and 54 shared no homology with any current database (**Table S9**), suggesting that many genes unique to *F. velutipes* were specifically expressed in fruiting bodies. This implied that many specific traits (species or clade specific) exclusively develop in fruiting bodies. A similar trend was observed in *S*. *commune* [2] and *Agaricus bisporus* [35].

### Lignocellulolysis

The *F*. *velutipes* genome contains a vast array of genes coding for the initial lignin degradation (Auxiliary Activities, AA; formerly FOLymes) [36], [37]. Among 69 AA genes in the *F*. *velutipes* genome, AA3 (glucose-methanol-choline oxidoreductase including cellobiose dehydrogenase, aryl-alcohol oxidase/glucose oxidase, alcohol oxidase, pyranose oxidase), AA7 (glucooligosaccharide oxidase), and AA9 (lytic polysaccharide monooxygenase;GH61) genes were prominently present, with 23, 15, and 16 genes, respectively (**Table S10**). These numbers were significantly higher than those in the closest known white-rot basidiomycete *S. commune* and *Phanerochaete chrysosporium*, with the exception of AA9 in *S. commune* (**Table 3**) [36]. Though *F. velutipes* lacked genes encoding AA10 (lytic polysaccharide monooxygenase; CBM33), there was a diverse presence of fungal ligninolytic enzymes (more than in *Postia placenta* [26] *L*. *bicolor* [23], *S. commune* [2], and *P. chrysosporium* [25]; **Table 3**), demonstrating a strongly developed lignin-degrading capacity. Furthermore, *F. velutipes* and eight other fungal species had a higher average number of AA9 enzymes (12) than those of other AA families (0∼8). AA9 family includes lytic polysaccharide monooxygenases, which were previously classified as GH61 family glycoside hydrolases. Moreover, AA9 enzymes have been recently characterized as copper-dependent polysaccharide monooxygenases [36]. A number of AA9 members have been identified mainly in fungal genomes, indicating the widespread distribution of this family of enzymes, especially in fungal wood decaying organisms. In this study, different AAs showed varying expression levels, with 11 out of 69 genes showing a 2-fold increase compared to the next highest expression level for a particular stage or tissue (**Table S10**, log2 values). Thus, the stipe and pileus, belonging to the same mature fruiting body, exhibited differential regulation of these enzyme groups, with overall expression being higher in the stipe than in the pileus (**Table S10**). The expression levels of glucose-methanol-choline (GMC) oxidoreductase (AA3) differed between the mycelium and fruiting body developmental stages (primordia, stipe, pileus). Additionally, because 4 out of the 11 genes upregulated by 2-fold or more exhibited peaked expression (≥5.5 log2 values) during the mycelium stage, AA3 appeared to have an important role in this stage. In addition, 6 AA genes (1 AA1, 1 AA2, 2 AA3 and 2 AA5) showed higher expression levels than the overall average expression level of the other AA genes during all stages and in all tissues (**Figure 3c** **and Table S10)**. Comparison of stipe tissue, pilei, and primordia further indicated a certain level of coregulation in stipes and pilei, i.e., expression in both the pileus and stipe was considerably lower or higher than in primordia. Expression analysis showed that all enzymes except for 1 AA3 were expressed in multiple stages, some with high expression connected to 1 specific stage. This, together with the sequential nature of degradation in *F. velutipes*, offers attractive possibilities for detailed studies on lignin degradation.

**Table 3:** Comparison of the number of AAs and CAZymes of *F*. *velutipes* with those of other fungi [36], [38].

		White rot fungi					Brown rot fungi					
		*F. velutipes*	*S. commune*	*P. chrysosporium*	*C. cinerea*		*P. placenta*	*L. bicolor*	*C. neofomans*	*U. maydis*		*N. crassa*
	AA1	3	2	1	17		3	12	3	1		10
	AA2	3	0	16	1		0	1	0	0		0
	AA3	23	7	8	25		7	5	0	1		3
	AA4	0	0	0	0		0	0	0	0		0
AAs	AA5	6	2	7	6		2	11	3	4		2
	AA6	2	4	4	3		1	2	2	1		1
	AA7	15	4	0	2		0	1	0	0		2
	AA8	1	3	2	6		0	0	0	0		8
	AA9	16	22	15	33		2	13	1	0		14
	AA10	0	0	0	0		0	0	0	1		0
Total		69	44	53	93		15	45	9	8		40
	GH	193	240	181	211		124	163	75	101		173
	GT	85	75	66	71		51	88	64	64		76
CAZymes	PL	23	16	4	13		4	7	3	1		4
	CE	91	30	20	54		13	20	8	19		23
Total		392	361	271	349		192	278	150	185		276

AA1 (laccases, ferroxidase, laccase-like multicopper oxidase), AA2 (manganase peroxidase, lignin peroxidase, versatile peroxidase), AA3 (glucose-methanol-choline oxidoreductase including cellobiose dehydrogenase, aryl-alcohol oxidase/glucose oxidase, alcohol oxidase, pyranose oxidase), AA4 (vanillyl alcohol oxidase), AA5 (glyoxal oxidase, galatose oxidase), AA6 (1,4-benzoquinone reductase), AA7 (glucooligosaccharide oxidase), AA8 (iron reductase domain), AA9 (lytic polysaccharide monooxygenase;GH61), AA10 (lytic polysaccharide monooxygenase; CBM33), GH (glycoside hydrolases); GT (glycosyl transferases), PL (polysaccharide lyases), CE (carbohydrate esterases).

The dbCAN CAZyme annotation of the predicted amino acid sequences of *F. velutipes* genes against the CAZy revealed 432 CAZymes, including 193 GHs, 85 GTs, 23 PLs, 91 CEs, and 40 CBMs, as well as multiple domain proteins, in the *F*. *velutipes* genome (**Table 3** **, S11, and S12**) [38]. Comparisons with the seven other basidiomycetes showed that *F. velutipes* had a higher number of all CAZymes (GHs, GTs, PLs, and CEs) than the average number for this lineage (162, 71, 8, and 30, respectively) [39]. In addition, *F. velutipes* had the highest number of genes encoding PLs (23) and CEs (91) among the seven other basidiomycetes. Comparisons with the 17 major CAZyme families reported in Floudas et al. [40] showed 69 genes in 14 of the 17 gene families. Absence of GH51 (endoglucanase) and CE9 (N-acetylglucosamine 6-phosphate deacetylase) is unusual in other white-rot fungi, but does not seem to impair the wood degrading ability of *F. velutipes* [41]. The distribution of CAZymes, with multiple copies in GH5, GH16, and GH18 (cellulose, β-glucan, and chitin degradation, respectively) families was consistent with other white-rot fungi, as was the presence of GH11 (xylanase) and CE12 (acetylesterase) gene families, which are generally absent in brown-rot fungi [40]. Cellulose is the main polymeric component of the plant cell wall and contains both highly crystalline regions and less-ordered amorphous regions. Glycoside hydrolase (GH) families GH6 and GH7 include cellobiohydrolases that are involved in the attack of crystalline cellulose (insoluble substrate) [42]. *F. velutipes* also contained 2 and 3 putative cellobiohydrolases assigned to family GH6 (absence in the 7 other basidiomycetes) and GH7, respectively (**Table S11**) [38].

RNA-seq analysis suggested stage- or tissue-restricted expression for some of these genes, since 13 and 2 out of 392 genes showed no expression in the mycelia stage and stipe, respectively (**Table S12**). Among the 14 genes showing no expression in the mycelia stage, GH71 and GH43 showed the highest expression levels during fruit body and primordial stage, respectively. The signal peptide prediction of amino acid sequences of *F. velutipes* against the SignalP 4.1 server (http://www.cbs.dtu.dk/services/SignalP/) [43] revealed 956 genes, including 38 AAs and 172 CAZymes, comprising the signal peptides (**Tables S10, S12, and S13**). Among the 172 CAZyme genes containing signal peptides, 11 genes (3 PL14, 1 PL3, 1 CE4, 1 CE15, 1 GH12, and 1 GH16, 2 GH43 and 1 GH71) and 3 genes (2 PL1 and 1 CBM) were not expressed at the mycelia stage (or fruit body) and both the fruit body and primordial stages (or mycelia), respectively. In addition, GH71 and CBM13 genes showed the highest expression levels during the fruit body and mycelia stages, respectively. Likewise, among the 38 AAs, one AA9 was not expressed at the mycelia stage. However, five AAs (1 AA1, 1 AA3, 2 AA5 and 1 AA9) showed higher expression levels at all stages than the other AA genes, including 33 AAs comprising signal peptides (**Tables S10 and S12**). The key step for conversion of lignocellulosic biomass into fermentable sugars is represented by the hydrolysis of polysaccharides, resulting from biomass pretreatment, by cellulases and hemicellulases. Filamentous fungi are the major source of cellulases and hemicellulases. The mechanisms regulating cellulase and hemicellulase genes have been studied in filamentous fungi (mainly in *Aspergillus* and *Trichoderma*) [44], [45], [46], and research on the regulation of cellulase and hemicellulase gene expression may be very useful for increasing production of these enzymes. In the hydrolysis process of cellulolytic residues by *T. reesei*, cellobiose is produced and accumulates, which inhibits further cellulases production. *T. reesei* β-glucosidase, which hydrolyzes cellobiose to glucose, has been described as having transglycosylation activity in the presence of insoluble substrates (crystalline cellulose), and is therefore possibly involved in cellulase gene induction [47]. *F. velutipes* also contained 6 putative β-glucosidase assigned to family GH1 (2) and GH3 (4) (**Table S5**). Among the 6 genes, 2 GH3 were gradually increased their expression levels through the developmental stages (from mycelia to fruit body) of *F. velutipes*. The genomes of both fungi (*A. niger* and *T. reesei*) encode many carbohydrate active enzymes (CAZy) which, through synergism, catalyse the deconstruction of cellulose and hemicelluloses, the main polysaccharides of lignocellulose. Regulation of the genes encoding these enzymes is governed by several transcription factors, including the activator XlnR/XYR1 (*A. nidulans*/*T. reesei*) and the carbon catabolite repressor CreA/CRE1 (*A. nidulans*/*T. reesei*) [48]. Foreman et al. [49] performed investigations on regulation of cellulase and hemicellulase genes expression in *T. reesei* by microarrays, five of which have been far identified to date: the positive regulators XYR1, ACE2, and the HAP2/3/5 complex; the repressor ACE1; and the carbon catabolite repressor CRE1 [47]. In addition, it has been reported that *creA*, *creB*, and *creC* genes products are involved in the regulatory mechanism of carbon catabolite repression in *Aspergillus* spp. [50], [51], [52], [53], [54]. *F. velutipes* was found to possess 681 putative transcription factors, including 7 homologs for regulation of (hemi)cellulolytic genes expression (1 HAP3, 3 HAP5, 1 PacC, 1 ACE1, and 1 CREC; **Table S7**).

### Alcohol Dehydrogenase Genes

*F. velutipes* has been reported to produce ethanol from complex substrates like whole crop sorghums and high-cellulose substrates as well as from defined monosugars, disugars, and oligosaccharides with high theoretical recovery rates up to 88% (similar to *S*. *cerevisiae*) [6], [55]. The *F. velutipes* genome indeed showed a strong potential for alcohol conversion, encoding 58 homologs to alcohol dehydrogenase genes covering a broad range of possible substrates (**Table S14**). Expression analysis showed that a large proportion of those enzymes were active in the mycelium. This, together with preferred degradation of lignin followed by (hemi)cellulose, makes *F. velutipes* a valuable candidate for CBP, allowing the combination of lignocellulose conversion to polysaccharides, hydrolysis of polysaccharides to sugars, and fermentation of (hexose and pentose) sugars to alcohol using a single organism. The identified alcohol dehydrogenases can now be easily characterized.

### Mushroom Formation

**Figure 4:**
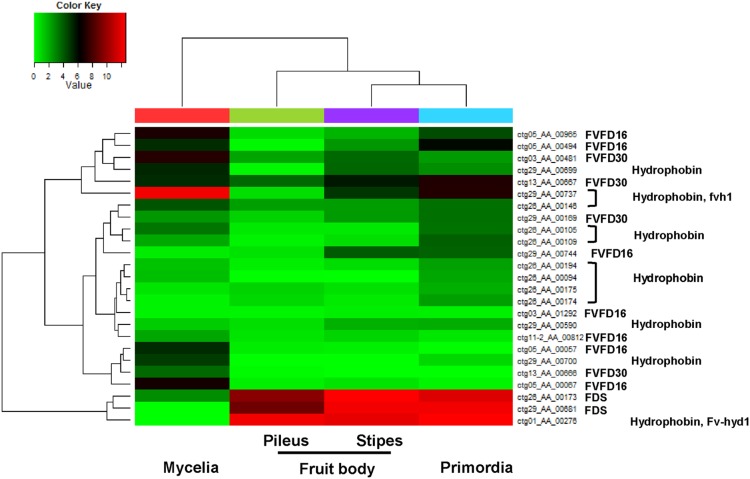
Heat map of expression of hydrophobin, *FVFD16*, *FVFD30*, and *FDS* genes at 3 developmental stages and in different tissues of *F*. *velutipes*. The bar at the top of the panel represents the expression values (log2 based normalization value). Detailed information is given in Table S15.

Mushroom formation was proposed to start with the formation of small aggregates from mycelium, formation of primordia, and outgrowth to mature fruiting bodies [56]. Studies on the development of *F. velutipes* have led to the identification of multiple environmental factors that trigger the formation of aggregates and primordia and influence the subsequent development of the mushroom. Comparative studies between different developmental steps, tissues, or environmental conditions on protein and gene expression [30], [57], [58], as well as studies on individual genes [59], [60] have provided several insights on mushroom development, but have not elucidated their complex global mechanisms and interactions. The genome sequence of *F. velutipes* revealed a series of genes associated with mushroom formation. Mating type genes [61], new hydrophobins (in addition to *FvHyd1*) [59], [60] (**Table S15**), and fruiting body-specific genes (*FDS*, *FVFD16*, and *FVFD30*), with 1, 5, and 3 putative homologs, respectively, were identified, and RNA-seq analysis suggested stage- or tissue-restricted expression for some of these genes (**Figure 4** **, Table S15, and Figure S2**). Interestingly, only 3 mitochondrial genes (*ATP9*, *SSU*, and *LSU*) were strongly expressed in *F. velutipes*, with *ATP9* lacking strong expression in the mycelium, suggesting that mitochondrial genes could be independently regulated in coordination with certain stages (**Table S8**).

### Mitochondrial DNA

**Figure 5:**
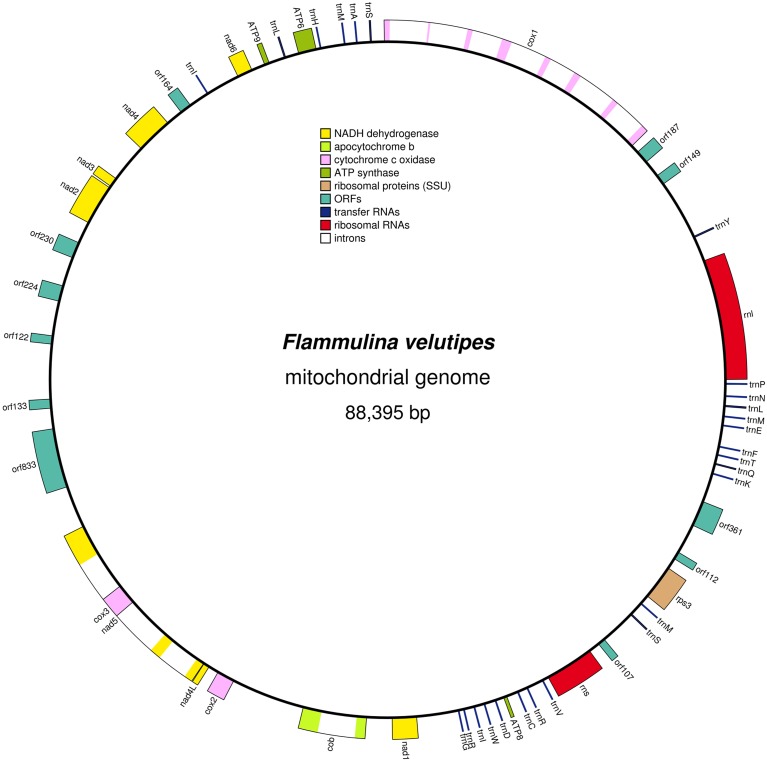
Mitochondrial genome map of *F. velutipes*.

Early during assembly, an additional scaffold harboring high AT content (>70%) was acquired. This scaffold was identified to be the mitochondrial DNA sequence (**Figure 5**), which was recently published for this strain (KACC42780 is 4019–20 as used in Yoon et al. [62]). Since mitochondrial DNA experiences a faster rate of mutation than nuclear DNA [63], mitochondrial sequences are of interest for strain identification and barcoding [64]. The sequence reported here could serve as a reference for *F. velutipes* strains. As expected, analysis showed little difference between our mtDNA (accession no. JF799107, 88,395 bp, and 83.46% AT content) and the previously published sequence (88,508 bp and 83.50% AT content). The same genes and tRNAs (26) were identified in a similar order, although 18 putative ORFs were predicted in our scaffold, while only 16 were reported by in Yoon et al. [62], likely resulting from differences in the interpretation of ORFs. *Cytochrome b* (*COB*) and *COX1* are always present in mitochondrial genomes [65], whereas most other genes are either in the mitochondrial DNA or in the organism’s genome. ATP9 has been reported to be present in both the mitochondrial and fungal genome of several species [66], but has clearly remained unique to the mitochondrial DNA in *F. velutipes*. As reported by Yoon et al. [62], the mtDNA of *F*. *velutipes* has a relatively low GC content (16.54% in our scaffold) in comparison to other basidiomycetes (21%–31%) [67], [68], and no origin of replication (ORI) could be detected. Being the first reported mtDNA within the Physalacriaceae, it will be interesting to see if these features are characteristic for this group or specific for *F. velutipes*. The absence of an ORI in mtDNA has also been reported in *Trametes cingulata* [69].

### Repeat Contents

Repeat sequences are thought to be important for generating genetic diversity and a consequence of evolution. RepeatMasker (http://repeatmasker.org) and Censor [70] were used to determine the copy numbers and chromosomal distributions of repetitive DNA of the *F*. *velutipes* genome. When we analyzed the repetitive elements by using RepeatMasker, various multicopy repetitive DNA regions (total of 93.5 kb) were identified in the *F*. *velutipes* genome. However, relatively low repeat DNA regions (0.263% of the genome: simple repeat, 0.1%; short interspersed nucleotide elements [SINEs], 0.003%; long interspersed nucleotide elements [LINEs], 0.011%; low complexity sequences, 0.085%; unclassified, 0.001%; LTRs, 0.001%, small RNAs, 0.034%) were found in the 35.6-Mb assembled genome of *F*. *velutipes* (**Table S16**).

Since retrotransposons are able to transcribe themselves via reverse transcription, they are often the major contributors to the repetitive contents in the genome [71]. The assembled genome of *F. velutipes* consisted of 10% (3,567,850 bp) repeat content, with the largest proportion comprising LTR retrotransposons (18%; 640,707 bp), including LTR Copia (4.29%; 153,361 bp) and Gypsy transposons (11.8%; 423,698 bp; **Table S17**).

## Discussion

*F. velutipes* is consumed for its culinary qualities and is also known as a wood rotting fungus that can degrade lignocellulose. Recently, *F*. *velutipes* was found to be a good producer of ethanol. Thus, *F*. *velutipes* is a highly attractive model/source for studying enzymes capable of efficient degradation of lignocellulosic biomass for bioethanol production. Here, we reported the sequencing of the *F. velutipes* genome, contributing valuable information for the development of genetic tools for this important organism and stimulating overall research on mushrooms and their various applications. Gene modeling revealed that the total number of genes in *F. velutipes* was comparable to that of other sequenced basidiomycetes with similar genome sizes (**Table 2**). However, the average intron length of *F. velutipes* was relatively larger (180.4 bp) than those of other sequenced species (75–127 bp). We also performed gene prediction using the AUGUSTUS tool [72] with default parameters trained in *C*. *cinerea* (**Table S1**). When we used the AUGUSTUS tool for gene prediction, the average intron length of *F. velutipes* was similar (91.5 bp) to those of other sequenced species. In addition, gene prediction using the AUGUSTUS tool revealed the total number of genes in *F. velutipes* was relatively fewer (10,645, excluding 5,032 isoforms) than that of other sequenced basidiomycetes of similar genome size (**Tables 2** **and S1**). This is why we used several methods for gene prediction, including *ab initio* gene structure prediction (Fgenesh), a homology-based approach (Fgenesh+), and transcriptome-based gene identification (Cufflinks). However, further studies are needed for evaluating the differences in average intron length between *F. velutipes* and other sequenced fugal species.

Wood exhibits high resistance to chemical and biological degradation due to lignin [26], and wood degradation by white-rot fungi usually starts with the depolymerization of this compound. This results in highly reactive lignin radicals that in turn attack any nearby wood polymers, causing further degradation via diverse reactions [8], [73]. The removal of the lignin matrix renders cellulose and hemicellulose compounds susceptible to enzymes classified as carbohydrate active enzymes (CAZymes) [74] and facilitates the further degradation of wood. *F*. *velutipes* has been indicated to follow a pattern of sequential wood degradation [41], implying that most lignin is degraded prior to degradation of other wood components, in contrast to simultaneous degradation [74]. The *F*. *velutipes* genome and NGS-based RNA-Seq revealed a vast array of genes associated with lignin and carbohydrate degradation common to white rot fungi, which helped us characterize its substrate metabolism in more detail. In addition, expression analysis revealed that several genes, annotated as AAs, were upregulated. Interestingly, the expression levels of these genes were specific to each developmental stage of *F*. *velutipes*. Notably, we observed substantial differences in the total number of genes between *F*. *velutipe*s and the close model fungus *S*. *commune*, especially in lignin degrading enzymes. The newly uncovered wood degrading capacity of *F. velutipes* offers interesting possibilities for more detailed studies on either lignin or (hemi-) cellulose degradation in complex wood substrates. The mutual interest in wood degradation by the mushroom industry and (ligno-) cellulose biomass-related industries further increases the significance of *F. velutipes* as a new model. Consequently, this approach allows the identification of common and specific genes of the *F*. *velutipes* genome during development. Furthermore, the analysis of genes specifically expressed at 3 different developmental stages would help to understand the mechanisms of fruiting at the molecular level and could improve artificial cultivation of mushrooms of various kinds of commercially important basidiomycetes.

According to a previous study [6], [75], *F*. *velutipes* efficiently converts d-glucose to ethanol with a theoretical recovery rate of 88% (similar to the ability of *S*. *cerevisiae*). In addition, *F*. *velutipes* is able to convert not only sucrose, but also maltose, cellobiose, cellotriose, and cellotetraose to ethanol, with almost the same recovery rate as that of d-glucose. Interestingly, the highest average expression level of 58 alcohol dehydrogenase genes was observed during the mycelia stage. Thus, the expression levels of these genes were 2 and 3 times more than those observed in the fruit body and at the primordial stage, respectively. Alcohol dehydrogenases (ADHs) constitute a large family of enzymes responsible for the reversible oxidation of alcohols to aldehydes with the concomitant reduction of NAD^+^ or NADP^+^. These enzymes have been identified not only in yeasts, but also in several other eukaryotes and even prokaryotes. The ADHs of *S*. *cerevisiae* have been studied intensively for over half a century [76]. The genes encoding classical ADHs in *S. cerevisiae* include *ADH1*, *ADH2*, *ADH3*, *ADH4*, and *ADH5* [77], [78], [79], [80]. Other genes include *SFA1*, *BDH1* [81], *BDH2* (YAL061W), *SOR1* (YAL246C), and the recently proposed *ADH6* (YMR218C) and *ADH7* (YCR105W) [81], [82], [83], [84], [85]. *F. velutipes* was found to possess five alcohol dehydrogenase genes, which showed sequence similarity to ADHs of *S. ceraviae* (**Table S14**). Interestingly, all of these homologs showed high expression levels during all stages and all tissues; thus, we focused on these candidate genes for bioethanol production. Our results demonstrating the extensive range of alcohol converting enzymes in the *F. velutipes* genome support the strong potential of this wood degrader for (consolidated) bioethanol production. Moreover, the highest expression level of alcohol dehydrogenase genes at the mycelial stage suggested that bioethanol could be efficiently produced by mycelia with low-cost biomass processing.

One of the major difficulties for the improvement of production methods is a poor understanding of mushroom formation. Very few genes have been identified that control initiation, succeeding stages, and proliferation. Mating type genes are of the few genes that are fundamental in mushroom formation. Often referred to as master regulatory genes, they control the establishment of the dikaryotic stage [56]. In tetrapolar species like *F. velutipes*, the *A* locus encodes homeodomain proteins that regulate clamp cell formation, synchronized nuclear division, and septation, while the *B* locus encodes pheromone receptors and pheromones that direct clamp cell fusion [86]. More importantly, mushroom formation can only be induced after both the A and B pathways have been activated. In our previous report [61], the mat *A* locus was part of a ∼350 kb region that was highly syntenic within Agaricales and contained conserved recombination hot spots linked to separation events of the mat*A* subloci in *F. velutipes* and other fungi. The homeodomain 2 genes *FvHD2*-*1* and *FvHD2*-*2* were specific for the mat*A*3 locus, whereas the homeodomain 1 gene *FvHD1*-*1* was detected in mat*A*3 and mat*A*4 mating types. EST libraries of monokaryotic and dikaryotic mycelia (unpublished data) of strains KACC42780 (matA3matB3), KACC43777 (mat*A*4mat*B*4), and KACC43778 (dikaryon, mat*A*3*A*4mat*B*3*B*4) confirmed these respective linkages.

The cell walls of filamentous fungi have been investigated in several species of basidiomycota, and studies have reported that the major components of the cell wall are chitin and β-1,3-glucan with β-1,6-linked branches [87], [88], [89], [90]. During the filamentous fungal life cycle, the cell walls are synthesized, re-orientated, and lysed [91], [92]. Cell wall lysis and changes in the constituent polysaccharides are essential processes during fruiting body development in basidiomycota and in autolysis of pileus of fruiting bodies. Yamada et al. [93] reported that *Fv-pda*, a gene coding for chitin deacetylase (CDA, deacetylates chitin to chitosan in the fungal cell wall) is specifically expressed during fruiting body development of *F*. *velutipes*. In addition, RT-PCR revealed that the transcript could be detected from all tissues, including the pileus and stipe, but the transcript levels were higher in stipes than in the pileus. The findings of the current study are consistent with those of Yamada et al. [93]. *F. velutipes* was found to possess five putative CDA genes, including one homolog, which showed sequence similarity to *Fv-pda* (**Table S5**). RNA-seq revealed that one CDA gene (homolog to *Fv-pda*) was expressed at all stages and tissues, but the expression level was higher in the stipe than in the pileus. In addition, the other CDA genes were also specifically expressed at different tissues and stages of *F*. *velutipes* (**Table S3**). These results are consistent with a previous study by Yamada et al. [93] and suggested that CDA would play an important role in the formation of fungal cell walls of fruiting bodies through conversion of chitin to partially deacetylated chitosan.

In conclusion, this study aims to advance understanding of the fundamental and cellular processes of mushroom-forming basidiomycete fungi for commercial production and industrial use. Although the complexity of the respective culture media indicates a possible correlation between complexity and the number of expressed genes (high to low: *F. velutipes*, MCM and wheatbran-sawdust; *L. bicolor*, outside in association with trees; *A. bisporus*, compost; *S. commune*, defined minimal medium) there is currently no clear explanation for the exceptionally high expression levels in *F. velutipes*. This, combined with the knowledge that *F. velutipes* responds to many environmental stimuli that are present (in reduced) combinations in other mushrooms, makes *F. velutipes* an effective model for studies on mushroom developmental processes and sensory pathways.

## Supporting Information

Figure S1 **Strategy for genome sequencing of** ***F*** **.** ***velutipes*** **.**
(TIF)

Figure S2 **Semi-quantitative RT-PCR analysis. M, mycelia; Pr, primordia; P, pileus; S, stipe.**
(TIF)

Table S1 **Distribution of the number of genes and exons in the** ***F. velutipes*** **genome.**
(XLS)

Table S2 **Alignment of ESTs on 12,218 predicted genes of** ***F. velutipes*** **.**
(XLS)

Table S3 **Global transcriptome analysis at 3 different developmental stages and in different tissues of** ***F. velutipes*** **.** Differential expression significances were determined using DEGseq [94].
(XLS)

Table S4 **Predicted tRNA genes in the** ***F. velutipes*** **genome.**
(XLS)

Table S5 **BLAST search results of 12,218 predicted genes of** ***F. velutipes*** **against the NCBI-NR database.**
(XLS)

Table S6 **Inparalogs and orthologs of predicted genes of** ***F. velutipes*** **.**
(XLS)

Table S7 **Predicted transcription factors in the** ***F. velutipes*** **genome (red, putative transcription factors for regulation of (hemi)cellulolytic genes expression).**
(XLS)

Table S8 **Expression levels of annotated genes in the** ***F. velutipes*** **mitochondrial genome at 3 different developmental stages and in different tissues.**
(XLS)

Table S9 **Genes expressed only during the fruit body stage of** ***F. velutipes*** **.**
(XLS)

Table S10 **Expression of auxiliary activities (AA) at 3 different developmental stages and in different tissues of** ***F. velutipes*** **.**
(XLS)

Table S11 **Distribution of CAZymes in the** ***F. velutipes*** **genome and in other sequenced fungal genomes [36]** **.**
(XLS)

Table S12 **Expression of predicted CAZyme genes at three different developmental stages and in different tissues of** ***F. velutipes*** **.**
(XLS)

Table S13 **Predicted signal peptides of the** ***F. velutipes*** **genome.**
(XLS)

Table S14 **Expression of predicted alcohol dehydrogenase genes at 3 different developmental stages and in different tissues of** ***F. velutipes*** **.**
(XLS)

Table S15 **Expression of hydrophobin,** ***FVFD16*** **,** ***FVFD30*** **, and** ***FDS*** **genes at 3 different developmental stages and in different tissues of** ***F. velutipes*** **.**
(XLS)

Table S16 **Repeated sequence distribution of the** ***F. velutipes*** **genome.**
(XLS)

Table S17 **Transposable element distribution in the** ***F. velutipes*** **genome.**
(XLS)

## References

[1] Matheny PB, Curtis JM, Hofstetter V, Aime MC, Moncalvo JM, et al (2006) Major clades of Agaricales: a multilocus phylogenetic overview. Mycologia 98: 982–995. 17486974 10.3852/mycologia.98.6.982

[2] Ohm RA, de Jong JF, Lugones LG, Aerts A, Kothe E, et al (2010) Genome sequence of the model mushroom *Schizophyllum commune* . Nat Biotechnol 28: 957–963. 20622885 10.1038/nbt.1643

[3] Psurtseva N (2005) Modern Taxonomy and Medicinal Value of the *Flammulina* Mushrooms. Int J Medicinal Mushrooms 7: 449.

[4] Sakamoto Y (2010) Protein expression during *Flammulina velutipes* fruiting body formation. Mycoscience 51: 163–169.

[5] Maehara T, Yoshida M, Ito Y, Tomita S, Takabatake K, et al (2010) Development of a gene transfer system for the mycelia of *Flammulina velutipes* Fv-1 strain. Biosci Biotechnol Biochem 74: 1126–1128. 20460695 10.1271/bbb.100021

[6] Mizuno R, Ichinose H, Honda M, Takabatake K, Sotome I, et al (2009) Use of Whole Crop Sorghums as a Raw Material in Consolidated Bioprocessing Bioethanol Production Using *Flammulina velutipes* . Biosci Biotechnol Biochem 73: 1671–1673. 19584528 10.1271/bbb.90099

[7] van Zyl WH, Lynd LR, den Haan R, McBride JE (2007) Consolidated bioprocessing for bioethanol production using *Saccharomyces cerevisiae* . Adv Biochem Eng Biotechnol 108: 205–235. 17846725 10.1007/10_2007_061

[8] Leonowicz A, Matuszewska A, Luterek J, Ziegenhagen D, Wojtas-Wasilewska M, et al (1999) Biodegradation of lignin by white rot fungi. Fungal Genet Biol 27: 175–185. 10441443 10.1006/fgbi.1999.1150

[9] Okamura T, Ogata T, Minamimoto N, Takeno T, Noda H, et al (2001) Characteristics of wine produced by mushroom fermentation. Biosci Biotechnol Biochem 65: 1596–1600. 11515544 10.1271/bbb.65.1596

[10] Okamura T, Ogata T, Toyoda M, Tanaka M, Minamimoto N, et al (2000) Production of Sake by mushroom fermentation. Mushroom Science and Biotechnology 8: 109–114.

[11] Kong WS, Cho YH, Jhune CS, Yoo YB, Kim KH (2004) Breeding of *Flammulina velutipes* strains adaptable to elevated-temperature. Mycobiology 32: 11–16.

[12] Frijters A, Zhang Z, Van Damme M, Wang GL, Ronald P, et al (1997) Construction of a bacterial artificial chromosome library containing large *Eco R*I and *Hind*III genomic fragments of lettuce. Theor Appl Genet 94: 390–399.

[13] Soderlund C, Humphray S, Dunham A, French L (2000) Contigs built with fingerprints, markers, and FPC V4.7. Genome Res 10: 1772–1787. 11076862 10.1101/gr.gr-1375r PMC310962

[14] Park YJ, Kim JK, Kong WS, Song ES, Lee CS, et al (2010) Electrophoretic karyotyping and construction of a bacterial artificial chromosome library of the winter mushroom *Flammulina velutipes* . Microbiol Res 165: 321–328. 19720512 10.1016/j.micres.2009.06.003

[15] Quinlan AR, Hall IM (2010) BEDTools: a flexible suite of utilities for comparing genomic features. Bioinformatics 26: 841–842. 20110278 10.1093/bioinformatics/btq033 PMC2832824

[16] Ashburner M, Ball CA, Blake JA, Botstein D, Butler H, et al (2000) Gene ontology: tool for the unification of biology. The Gene Ontology Consortium. Nat Genet 25: 25–29. 10802651 10.1038/75556 PMC3037419

[17] Tatusov RL, Galperin MY, Natale DA, Koonin EV (2000) The COG database: a tool for genome-scale analysis of protein functions and evolution. Nucleic Acids Res 28: 33–36. 10592175 10.1093/nar/28.1.33 PMC102395

[18] Lowe TM, Eddy SR (1997) tRNAscan-SE: a program for improved detection of transfer RNA genes in genomic sequence. Nucleic Acids Res 25: 955–964. 9023104 10.1093/nar/25.5.955 PMC146525

[19] Li L, Stoeckert CJ Jr, Roos DS (2003) OrthoMCL: identification of ortholog groups for eukaryotic genomes. Genome Res 13: 2178–2189. 12952885 10.1101/gr.1224503 PMC403725

[20] Galagan JE, Calvo SE, Cuomo C, Ma LJ, Wortman JR, et al (2005) Sequencing of *Aspergillus nidulans* and comparative analysis with *A*. *fumigatus* and *A*. *oryzae* . Nature 438: 1105–1115. 16372000 10.1038/nature04341

[21] Stajich JE, Wilke SK, Ahren D, Au CH, Birren BW, et al (2010) Insights into evolution of multicellular fungi from the assembled chromosomes of the mushroom *Coprinopsis cinerea* (*Coprinus cinereus*). Proc Natl Acad Sci USA 107: 11889–11894. 20547848 10.1073/pnas.1003391107 PMC2900686

[22] Loftus BJ, Fung E, Roncaglia P, Rowley D, Amedeo P, et al (2005) The genome of the basidiomycetous yeast and human pathogen *Cryptococcus neoformans* . Science 307: 1321–1324. 15653466 10.1126/science.1103773 PMC3520129

[23] Martin F, Aerts A, Ahren D, Brun A, Danchin EG, et al (2008) The genome of *Laccaria bicolor* provides insights into mycorrhizal symbiosis. Nature 452: 88–92. 18322534 10.1038/nature06556

[24] Galagan JE, Calvo SE, Borkovich KA, Selker EU, Read ND, et al (2003) The genome sequence of the filamentous fungus *Neurospora crassa* . Nature 422: 859–868. 12712197 10.1038/nature01554

[25] Martinez D, Larrondo LF, Putnam N, Gelpke MD, Huang K, et al (2004) Genome sequence of the lignocellulose degrading fungus *Phanerochaete chrysosporium* strain RP78. Nat Biotechnol 22: 695–700. 15122302 10.1038/nbt967

[26] Martinez D, Challacombe J, Morgenstern I, Hibbett D, Schmoll M, et al (2009) Genome, transcriptome, and secretome analysis of wood decay fungus *Postia placenta* supports unique mechanisms of lignocellulose conversion. Proc Natl Acad Sci USA 106: 1954–1959. 19193860 10.1073/pnas.0809575106 PMC2644145

[27] Goffeau A, Barrell BG, Bussey H, Davis RW, Dujon B, et al. (1996) Life with 6000 genes. Science 274: 546, 563–547. 10.1126/science.274.5287.546 8849441

[28] Kamper J, Kahmann R, Bolker M, Ma LJ, Brefort T, et al (2006) Insights from the genome of the biotrophic fungal plant pathogen *Ustilago maydis* . Nature 444: 97–101. 17080091 10.1038/nature05248

[29] Yin Y, Mao X, Yang J, Chen X, Mao F, et al (2012) dbCAN: a web resource for automated carbohydrate-active enzyme annotation. Nucleic Acids Res 40: W445–451. 22645317 10.1093/nar/gks479 PMC3394287

[30] Joh JH, Kim KY, Lim JH, Son ES, Park HR, et al (2009) Comparative analysis of expressed sequence tags from *Flammulina velutipes* at different developmental stages. J Microbiol Biotechnol 19: 774–780. 19734714

[31] Li H, Durbin R (2009) Fast and accurate short read alignment with Burrows-Wheeler transform. Bioinformatics 25: 1754–1760. 19451168 10.1093/bioinformatics/btp324 PMC2705234

[32] McKenna A, Hanna M, Banks E, Sivachenko A, Cibulskis K, et al (2010) The Genome Analysis Toolkit: a MapReduce framework for analyzing next-generation DNA sequencing data. Genome Res 20: 1297–1303. 20644199 10.1101/gr.107524.110 PMC2928508

[33] Li H, Handsaker B, Wysoker A, Fennell T, Ruan J, et al (2009) The Sequence Alignment/Map format and SAMtools. Bioinformatics 25: 2078–2079. 19505943 10.1093/bioinformatics/btp352 PMC2723002

[34] Bolstad BM, Irizarry RA, Astrand M, Speed TP (2003) A comparison of normalization methods for high density oligonucleotide array data based on variance and bias. Bioinformatics 19: 185–193. 12538238 10.1093/bioinformatics/19.2.185

[35] Morin E, Kohler A, Baker AR, Foulongne-Oriol M, Lombard V, et al (2012) Genome sequence of the button mushroom *Agaricus bisporus* reveals mechanisms governing adaptation to a humic-rich ecological niche. Proc Natl Acad Sci USA 109: 17501–17506. 23045686 10.1073/pnas.1206847109 PMC3491501

[36] Levasseur A, Drula E, Lombard V, Coutinho PM, Henrissat B (2013) Expansion of the enzymatic repertoire of the CAZy database to integrate auxiliary redox enzymes. Biotechnol Biofuels 6: 41. 23514094 10.1186/1754-6834-6-41 PMC3620520

[37] Levasseur A, Piumi F, Coutinho PM, Rancurel C, Asther M, et al (2008) FOLy: an integrated database for the classification and functional annotation of fungal oxidoreductases potentially involved in the degradation of lignin and related aromatic compounds. Fungal Genet Biol 45: 638–645. 18308593 10.1016/j.fgb.2008.01.004

[38] Bao D, Gong M, Zheng H, Chen M, Zhang L, et al (2013) Sequencing and comparative analysis of the straw mushroom (*Volvariella volvacea*) genome. PLoS One 8: e58294. 23526973 10.1371/journal.pone.0058294 PMC3602538

[39] Chen B, Gui F, Xie B, Deng Y, Sun X, et al (2013) Composition and expression of genes encoding carbohydrate-active enzymes in the straw-degrading mushroom *Volvariella volvacea* . PLoS One 8: e58780. 23554925 10.1371/journal.pone.0058780 PMC3595290

[40] Floudas D, Binder M, Riley R, Barry K, Blanchette RA, et al (2012) The Paleozoic origin of enzymatic lignin decomposition reconstructed from 31 fungal genomes. Science 336: 1715–1719. 22745431 10.1126/science.1221748

[41] Pal M, Calvo A, Terron M, Gonzalez A (1995) Solid-state fermentation of sugarcane bagasse with *Flammulina velutipes* and *Trametes versicolor* . World J Microb Biot 11: 541–545. 10.1007/BF00286370 24414910

[42] Baldrian P, Valaskova V (2008) Degradation of cellulose by basidiomycetous fungi. FEMS Microbiol Rev 32: 501–521. 18371173 10.1111/j.1574-6976.2008.00106.x

[43] Petersen TN, Brunak S, von Heijne G, Nielsen H (2011) SignalP 4.0: discriminating signal peptides from transmembrane regions. Nat Methods 8: 785–786. 21959131 10.1038/nmeth.1701

[44] Noguchi Y, Sano M, Kanamaru K, Ko T, Takeuchi M, et al (2009) Genes regulated by AoXlnR, the xylanolytic and cellulolytic transcriptional regulator, in *Aspergillus oryzae* . Appl Microbiol Biotechnol 85: 141–154. 19777228 10.1007/s00253-009-2236-9

[45] Stricker AR, Mach RL, de Graaff LH (2008) Regulation of transcription of cellulases- and hemicellulases-encoding genes in *Aspergillus niger* and *Hypocrea jecorina* (*Trichoderma reesei*). Appl Microbiol Biotechnol 78: 211–220. 18197406 10.1007/s00253-007-1322-0

[46] van Peij NN, Visser J, de Graaff LH (1998) Isolation and analysis of xlnR, encoding a transcriptional activator co-ordinating xylanolytic expression in *Aspergillus niger.* . Mol Microbiol 27: 131–142. 9466262 10.1046/j.1365-2958.1998.00666.x

[47] Kubicek CP, Mikus M, Schuster A, Schmoll M, Seiboth B (2009) Metabolic engineering strategies for the improvement of cellulase production by *Hypocrea jecorina* . Biotechnol Biofuels 2: 19. 19723296 10.1186/1754-6834-2-19 PMC2749017

[48] Amore A, Giacobbe S, Faraco V (2013) Regulation of cellulase and hemicellulase gene expression in fungi. Curr Genomics 14: 230–249. 24294104 10.2174/1389202911314040002 PMC3731814

[49] Foreman PK, Brown D, Dankmeyer L, Dean R, Diener S, et al (2003) Transcriptional regulation of biomass-degrading enzymes in the filamentous fungus *Trichoderma reesei* . Journal of Biological Chemistry 278: 31988–31997. 12788920 10.1074/jbc.M304750200

[50] Arst HN Jr, Cove DJ (1973) Nitrogen metabolite repression in *Aspergillus nidulans* . Mol Gen Genet 126: 111–141. 4591376 10.1007/BF00330988

[51] Dowzer CE, Kelly JM (1991) Analysis of the creA gene, a regulator of carbon catabolite repression in *Aspergillus nidulans* . Mol Cell Biol 11: 5701–5709. 1922072 10.1128/mcb.11.11.5701-5709.1991 PMC361941

[52] Hynes MJ, Kelly JM (1977) Pleiotropic mutants of *Aspergillus nidulans* altered in carbon metabolism. Mol Gen Genet 150: 193–204. 320455 10.1007/BF00695399

[53] Lockington RA, Kelly JM (2001) Carbon catabolite repression in *Aspergillus nidulans* involves deubiquitination. Molecular Microbiology 40: 1311–1321. 11442830 10.1046/j.1365-2958.2001.02474.x

[54] Todd RB, Lockington RA, Kelly JM (2000) The Aspergillus nidulans *creC* gene involved in carbon catabolite repression encodes a WD40 repeat protein. Mol Gen Genet 263: 561–570. 10852476 10.1007/s004380051202

[55] Maehara T, Ichinose H, Furukawa T, Ogasawara W, Takabatake K, et al (2013) Ethanol production from high cellulose concentration by the basidiomycete fungus *Flammulina velutipes* . Fungal Biol 117: 220–226. 23537879 10.1016/j.funbio.2013.02.002

[56] Kues U (2000) Life history and developmental processes in the basidiomycete *Coprinus cinereus* . Microbiol Mol Biol Rev 64: 316–353. 10839819 10.1128/mmbr.64.2.316-353.2000 PMC98996

[57] Sakamoto Y, Ando A, Tamai Y, Miura K, Yajima T (2002) Protein expressions during fruit body induction of *Flammulina velutipes* under reduced temperature. Mycological Research 106: 222–227.

[58] Sakamoto Y, Ando A, Tamai Y, Yajima T (2007) Pileus differentiation and pileus-specific protein expression in *Flammulina velutipes* . Fungal Genetics and Biology 44: 14–24. 16877016 10.1016/j.fgb.2006.06.002

[59] Ando A, Harada A, Miura K, Tamai Y (2001) A gene encoding a hydrophobin, *fvh1*, is specifically expressed after the induction of fruiting in the edible mushroom *Flammulina velutipes* . Current Genetics 39: 190–197. 11409181 10.1007/s002940100193

[60] Yamada M, Sakuraba S, Shibata K, Inatomi S, Okazaki M, et al (2005) Cloning and characterization of a gene coding for a hydrophobin, *Fv-hyd1*, specifically expressed during fruiting body development in the basidiomycete *Flammulina velutipes* . Applied Microbiology and Biotechnology 67: 240–246. 15834718 10.1007/s00253-004-1776-2

[61] van Peer AF, Park SY, Shin PG, Jang KY, Yoo YB, et al (2011) Comparative genomics of the mating-type loci of the mushroom *Flammulina velutipes* reveals widespread synteny and recent inversions. PLoS One 6: e22249. 21799803 10.1371/journal.pone.0022249 PMC3140503

[62] Yoon H, You YH, Woo JR, Park YJ, Kong WS, et al (2012) The mitochondrial genome of the white-rot fungus *Flammulina velutipes* . J Gen Appl Microbiol 58: 331–337. 22990494 10.2323/jgam.58.331

[63] Bruns T, Szaro T (1992) Rate and mode differences between nuclear and mitochondrial small-subunit rRNA genes in mushrooms. Mol Biol Evol 9: 836–855. 1382179 10.1093/oxfordjournals.molbev.a040760

[64] Mouhamadou B, Carriconde F, Gryta H, Jargeat P, Manzi S, et al (2008) Molecular evolution of mitochondrial ribosomal DNA in the fungal genus *Tricholoma*: barcoding implications. Fungal Genet Biol 45: 1219–1226. 18647655 10.1016/j.fgb.2008.06.006

[65] Gray MW (2012) Mitochondrial evolution. Cold Spring Harb Perspect Biol 4: a011403. 22952398 10.1101/cshperspect.a011403 PMC3428767

[66] Dequard-Chablat M, Sellem CH, Golik P, Bidard F, Martos A, et al (2011) Two nuclear life cycle-regulated genes encode interchangeable subunits c of mitochondrial ATP synthase in *Podospora anserina* . Mol Biol Evol 28: 2063–2075. 21273631 10.1093/molbev/msr025

[67] Formighieri EF, Tiburcio RA, Armas ED, Medrano FJ, Shimo H, et al (2008) The mitochondrial genome of the phytopathogenic basidiomycete *Moniliophthora perniciosa* is 109 kb in size and contains a stable integrated plasmid. Mycological Research 112: 1136–1152. 18786820 10.1016/j.mycres.2008.04.014

[68] Wang Y, Zeng F, Hon CC, Zhang Y, Leung FC (2008) The mitochondrial genome of the Basidiomycete fungus *Pleurotus ostreatus* (oyster mushroom). FEMS Microbiol Lett 280: 34–41. 18248422 10.1111/j.1574-6968.2007.01048.x

[69] Haridas S, Gantt JS (2010) The mitochondrial genome of the wood-degrading basidiomycete *Trametes cingulata* . FEMS Microbiol Lett 308: 29–34. 20455947 10.1111/j.1574-6968.2010.01979.x

[70] Kohany O, Gentles AJ, Hankus L, Jurka J (2006) Annotation, submission and screening of repetitive elements in Repbase: RepbaseSubmitter and Censor. BMC Bioinformatics 7: 474. 17064419 10.1186/1471-2105-7-474 PMC1634758

[71] Santana MF, Silva JC, Batista AD, Ribeiro LE, da Silva GF, et al (2012) Abundance, distribution and potential impact of transposable elements in the genome of *Mycosphaerella fijiensis* . BMC Genomics 13: 720. 23260030 10.1186/1471-2164-13-720 PMC3562529

[72] Stanke M, Morgenstern B (2005) AUGUSTUS: a web server for gene prediction in eukaryotes that allows user-defined constraints. Nucleic Acids Research 33: W465–W467. 15980513 10.1093/nar/gki458 PMC1160219

[73] Guillén F, Martínez MJ, Gutiérrez A (2005) Biodegradation of lignocellu-losics: microbial, chemical, and enzymatic aspects of the fungal attack of lignin. International Microbiology 8: 195–204. 16200498

[74] Cantarel BL, Coutinho PM, Rancurel C, Bernard T, Lombard V, et al (2009) The Carbohydrate-Active EnZymes database (CAZy): an expert resource for Glycogenomics. Nucleic Acids Res 37: D233–238. 18838391 10.1093/nar/gkn663 PMC2686590

[75] Mizuno R, Ichinose H, Maehara T, Takabatake K, Kaneko S (2009) Properties of ethanol fermentation by *Flammulina velutipes* . Biosci Biotechnol Biochem 73: 2240–2245. 19809184 10.1271/bbb.90332

[76] de Smidt O, du Preez JC, Albertyn J (2008) The alcohol dehydrogenases of *Saccharomyces cerevisiae*: a comprehensive review. FEMS Yeast Res 8: 967–978. 18479436 10.1111/j.1567-1364.2008.00387.x

[77] Ciriacy M (1975) Genetics of alcohol dehydrogenase in *Saccharomyces cerevisiae*. II. Two loci controlling synthesis of the glucose-repressible ADH II. Mol Gen Genet 138: 157–164. 1105150 10.1007/BF02428119

[78] Feldmann H, Aigle M, Aljinovic G, Andre B, Baclet MC, et al (1994) Complete DNA sequence of yeast chromosome II. EMBO J 13: 5795–5809. 7813418 10.1002/j.1460-2075.1994.tb06923.x PMC395553

[79] Lutstorf U, Megnet R (1968) Multiple forms of alcohol dehydrogenase in *Saccharomyces cerevisiae.* I. Physiological control of ADH-2 and properties of ADH-2 and ADH-4. Arch Biochem Biophys 126: 933–944. 5686604 10.1016/0003-9861(68)90487-6

[80] Walton JD, Paquin CE, Kaneko K, Williamson VM (1986) Resistance to antimycin A in yeast by amplification of ADH4 on a linear, 42 kb palindromic plasmid. Cell 46: 857–863. 3019553 10.1016/0092-8674(86)90067-x

[81] Gonzalez E, Fernandez MR, Larroy C, Sola L, Pericas MA, et al (2000) Characterization of a (2R,3R)-2,3-butanediol dehydrogenase as the *Saccharomyces cerevisiae* YAL060W gene product - Disruption and induction of the gene. Journal of Biological Chemistry 275: 35876–35885. 10938079 10.1074/jbc.M003035200

[82] Larroy C, Fernandez MR, Gonzalez E, Pares X, Biosca JA (2002) Characterization of the *Saccharomyces cerevisiae* YMR318C (ADH6) gene product as a broad specificity NADPH-dependent alcohol dehydrogenase: relevance in aldehyde reduction. Biochemical Journal 361: 163–172. 11742541 10.1042/0264-6021:3610163 PMC1222291

[83] Larroy C, Pares X, Biosca JA (2002) Characterization of a *Saccharomyces cerevisiae* NADP(H)-dependent alcohol dehydrogenase (ADHVII), a member of the cinnamyl alcohol dehydrogenase family. European Journal of Biochemistry 269: 5738–5745. 12423374 10.1046/j.1432-1033.2002.03296.x

[84] van Iersel MF, Eppink MH, van Berkel WJ, Rombouts FM, Abee T (1997) Purification and characterization of a novel NADP-dependent branched-chain alcohol dehydrogenase from *Saccharomyces cerevisiae* . Appl Environ Microbiol 63: 4079–4082. 9327572 10.1128/aem.63.10.4079-4082.1997 PMC168719

[85] Wehner EP, Rao E, Brendel M (1993) Molecular structure and genetic regulation of SFA, a gene responsible for resistance to formaldehyde in *Saccharomyces cerevisiae*, and characterization of its protein product. Mol Gen Genet 237: 351–358. 8483449 10.1007/BF00279438

[86] Raudaskoski M, Kothe E (2010) Basidiomycete mating type genes and pheromone signaling. Eukaryot Cell 9: 847–859. 20190072 10.1128/EC.00319-09 PMC2901643

[87] Bottom CB, Siehr DJ (1979) Structure of an alkali-soluble polysaccharide from the hyphal wall of the basidiomycete *Coprinus macrorhizus* var. *microsporus* . Carbohydrate Research 77: 169–181.

[88] Mol PC, Wessels JGH (1990) Differences in wall structure between substrate hyphae and hyphae of fruit-body stipes in *Agaricus bisporus* . Mycological Research 94: 472–479.

[89] Shida M, Ushioda Y, Nakajima T, Matsuda K (1981) Structure of the alkali-insoluble skeletal glucan of Lentinus edodes. J Biochem 90: 1093–1100. 7198117 10.1093/oxfordjournals.jbchem.a133561

[90] Wessels JG, Kreger DR, Marchant R, Regensburg BA, De Vries OM (1972) Chemical and morphological characterization of the hyphal wall surface of the basidiomycete *Schizophyllum commune* . Biochim Biophys Acta 273: 346–358. 5080323 10.1016/0304-4165(72)90226-7

[91] Moore D (1998) Fungal morphogenesis. Cambridge; New York: Cambridge University Press. xiv, 469 p.

[92] Wessels JG (1993) Fruiting in the higher fungi. Adv Microb Physiol 34: 147–202. 8452092 10.1016/s0065-2911(08)60029-6

[93] Yamada M, Kurano M, Inatomi S, Taguchi G, Okazaki M, et al (2008) Isolation and characterization of a gene coding for chitin deacetylase specifically expressed during fruiting body development in the basidiomycete *Flammulina velutipes* and its expression in the yeast *Pichia pastoris* . FEMS Microbiol Lett 289: 130–137. 19054103 10.1111/j.1574-6968.2008.01361.x

[94] Wang L, Feng Z, Wang X, Wang X, Zhang X (2010) DEGseq: an R package for identifying differentially expressed genes from RNA-seq data. Bioinformatics 26: 136–138. 19855105 10.1093/bioinformatics/btp612

