# Supplementary figures and images for: Whole Genome and Global Gene Expression Analyses of the Model Mushroom Flammulina velutipes Reveal a High Capacity for Lignocellulose Degradation
Source: PLoS One. 2014 Apr 8;9(4):e93560. doi: 10.1371/journal.pone.0093560 (PMC3979922; doi:10.1371/journal.pone.0093560)

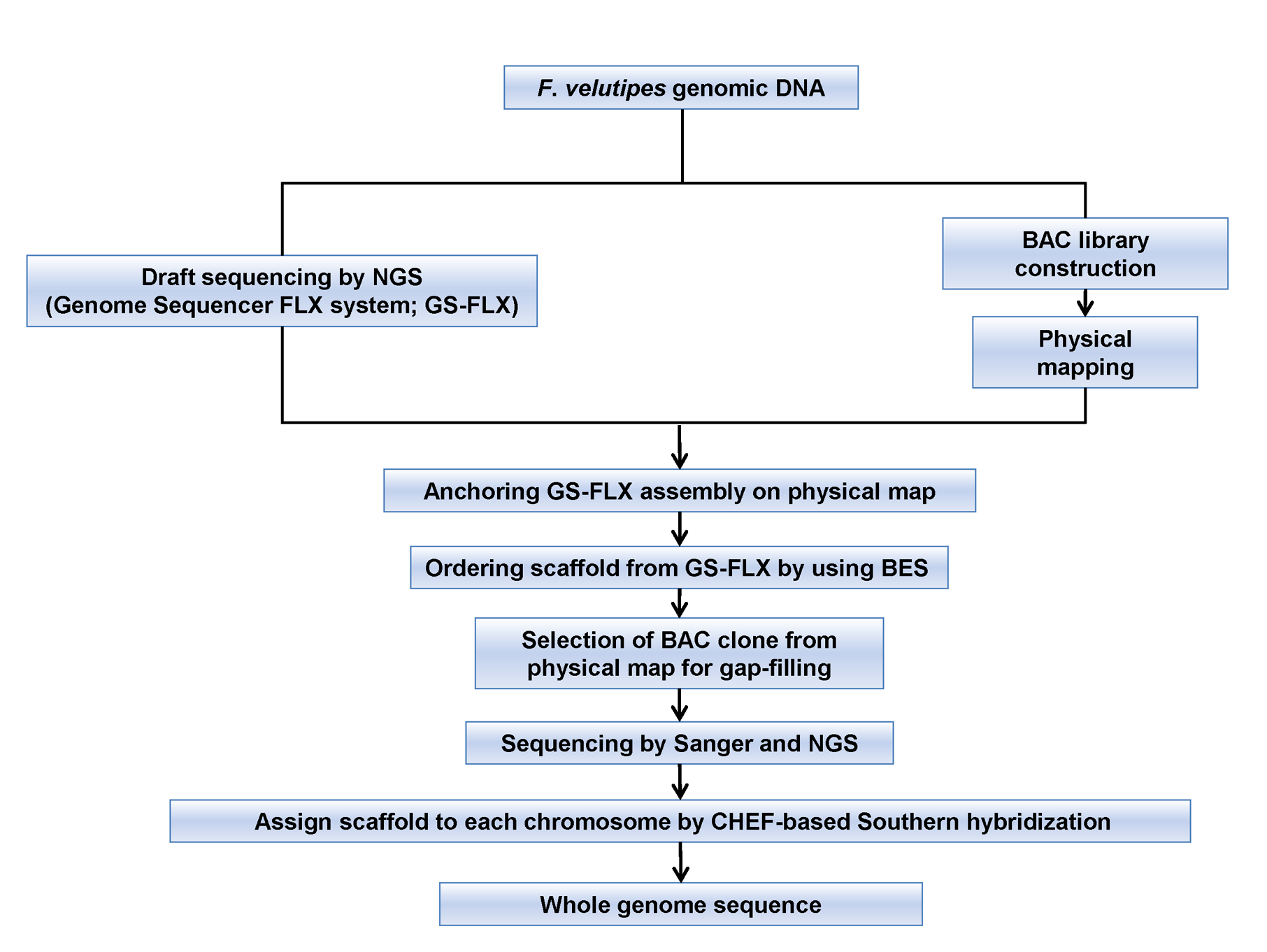

Supplement: Figure S1 — Strategy for genome sequencing of F . velutipes . (TIF) [file pone.0093560.s001.tif]

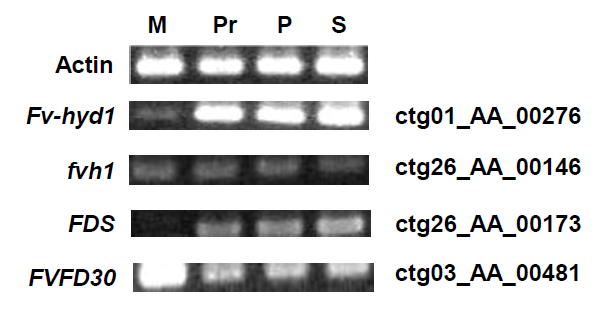

Supplement: Figure S2 — Semi-quantitative RT-PCR analysis. M, mycelia; Pr, primordia; P, pileus; S, stipe. (TIF) [file pone.0093560.s002.tif]
